# Case Report: Primary Mediastinal Large B-Cell Lymphoma Invasion of Extranodal Thyroid Tissue Mimicking Tuberculosis and Confounded by Similar Ultrasonic Appearance

**DOI:** 10.3389/fonc.2022.879295

**Published:** 2022-05-18

**Authors:** Ying Wang, Menghan Chen, Chen Ni, Jiahui Tong, Peijun Chen, Ying Zhang, Gaoyi Yang

**Affiliations:** ^1^ Department of Ultrasonography, School of Medicine, Hangzhou Normal University, Hangzhou, China; ^2^ Department of Ultrasonography, The Second Clinical Medical College, Zhejiang Chinese Medical University, Hangzhou, China; ^3^ Department of Ultrasonography, Affiliated Hangzhou Chest Hospital, Zhejiang University School of Medicine. Chinese and Western Hospital of Zhejiang Province (Hangzhou Red Cross Hospital), Hangzhou, China

**Keywords:** tuberculosis, contrast-enhanced ultrasound (CEUS), primary mediastinal large B-cell lymphoma (PMBCL), thyroid, ultrasound-guided puncture

## Abstract

**Background:**

Primary mediastinal large B-cell lymphoma (PMBCL) is a rare type of diffuse large B-cell lymphoma, which has significant features that overlap with those of Hodgkin’s lymphoma. Ultrasound is a commonly used modality to characterize superficial lymph no5des, and ultrasonic findings are often used to distinguish lymphoma from lymph node tuberculosis in daily clinical practice. Although a common malignancy, lymphoma rarely involves extranodal tissues.

**Case Presentation:**

Here we report the case of a 42-year-old Chinese male patient with PMBCL who was misdiagnosed with tuberculosis because of extranodal invasion. He visited our hospital for a neck mass that he had been noting for 1 week. Ultrasound revealed multiple enlarged lymph nodes on both sides of the neck. The lesions appeared to involve the surrounding soft tissue and thyroid gland, resembling a tuberculous sinus tract formation. Cervical spine computed tomography showed no obvious abnormalities in the cervical cone or bone damage. Contrast-enhanced ultrasound indicated that one of the enlarged lymph nodes in the right neck was rich in blood supply and exhibited centripetal enhancement, with uniform high enhancement at the peak. The patient underwent two ultrasound-guided punctures; the first puncture was performed for an enlarged lymph node in the right neck at Hangzhou Red Cross Hospital. Hodgkin’s lymphoma was suspected based on pathological and immunohistochemical findings, whereas a rare type of diffuse large B-cell lymphoma was suspected at Zhejiang Cancer Hospital.

**Conclusions:**

Lymphoma is often misdiagnosed, causing delayed treatment initiation and affecting patient outcomes as the disease progresses. The present case demonstrates that the ultrasonic appearance of lymphoma may sometimes be confused with that of tuberculosis. Although ultrasound-guided needle biopsy has a high diagnostic accuracy, it may also cause diagnostic deviation because of insufficient sampling volume. Moreover, owing to the enlargement of multiple lymph nodes due to lymphoma or lymph node tuberculosis, puncturing different lymph nodes may provide different results.

## Introduction

Primary mediastinal B-cell lymphoma (PMBCL) is an aggressive B-cell lymphoma of thymic origin and primarily noted in young women with a median age of 35 years at diagnosis. It accounts for approximately 2%–4% of all non-Hodgkin’s lymphoma and approximately 7% of diffuse large B-cell lymphomas (1, 2). More than two-thirds of patients with PMBCL present with a large anterior mediastinal mass with local tumor compression, leading to rapidly progressive clinical symptoms, such as dyspnea, cough, dysphagia, airway and great vessel compression, and superior vena cava obstruction (3). As a subtype of non-Hodgkin’s lymphoma, painless lymphadenopathy is also common, and some patients may have systemic symptoms, particularly night sweats, persistent fever, and unexplained weight loss (4). Despite increased awareness regarding the disease and advances in equipment and technology, the diagnosis of lymphoma remains a challenge. Lymph node tuberculosis is one of the diseases that should be differentiated from lymphoma.

Ultrasound is a common tool for characterizing superficial lymph nodes. It visualizes lymph node boundary, size, and blood flow, thereby differentiating among various causes of lymphadenopathy (lymphoma, tuberculosis, etc.). In daily clinical practice, ultrasonic characteristics are often used to distinguish lymphoma from lymph node tuberculosis. Although lymphoma is common, the involvement of extranodal tissue is rare in patients with lymphoma (5). This report describes the case of a patient who was admitted to our hospital and presented with symptoms similar to tuberculosis and but was later diagnosed with PMBCL. We describe the clinical progress of the patient from the initial diagnosis to improvement after treatment.

## Case Description

**Figure 1:**
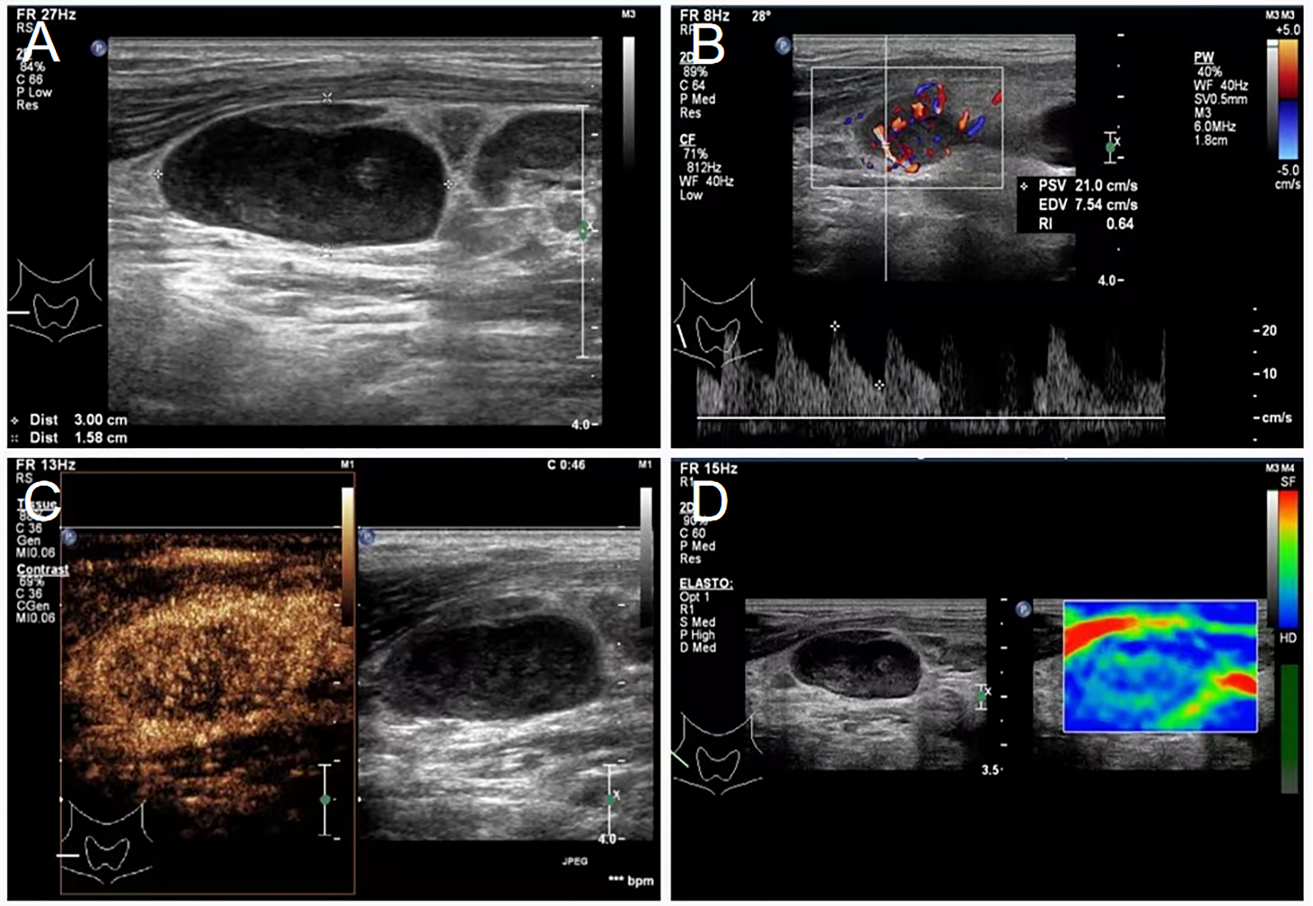
Lymphadenopathy noted on ultrasound in the neck of a 43-year-old male patient diagnosed with primary mediastinal B-cell lymphoma. **(A)** Sonogram revealing enlarged lymph nodes of the right neck, 3.0 × 1.6 cm in size, oval, uneven, and with a fair margin. **(B)** Color Doppler examination showing abundant blood flow in enlarged lymph nodes of the right neck, a lymphatic hilum, PSV: 21.0 cm/s, RI: 0.64. **(C)** Contrast-enhanced ultrasound showing abundant lymph node blood supply in area III of the right neck. **(D)** Shear-wave elastography of the lymph node, which is indicated by the mostly blue area. PSV, peak systolic velocity; RI, resistance index.

**Figure 2:**
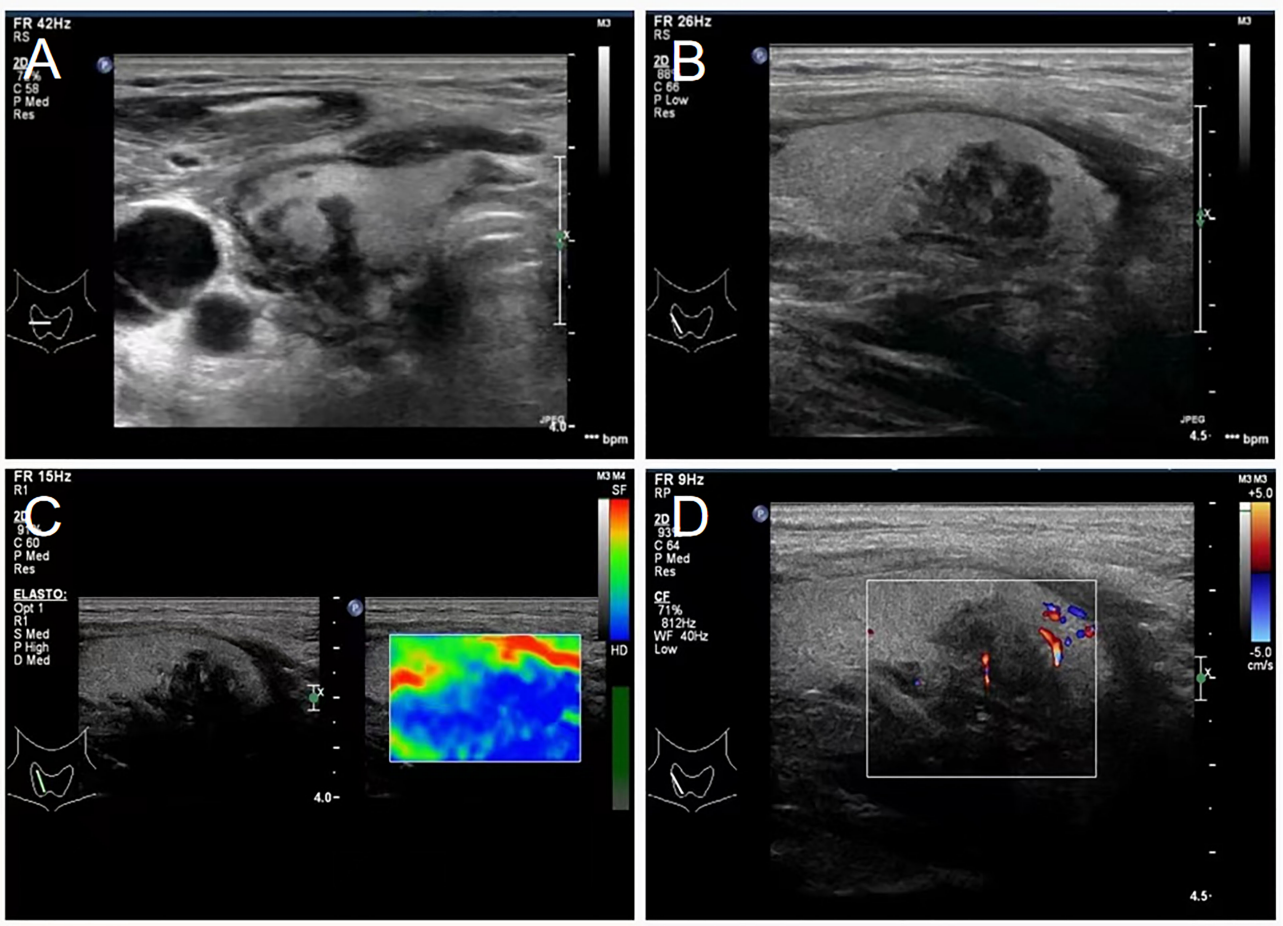
Transverse and longitudinal ultrasound showing thyroid invasion. **(A)** Ultrasound transects of thyroid showing a hypoechoic, irregular, and ill-defined thyroid at the right side. **(B)** Ultrasonography of the thyroid showing a low-echo area in the middle and lower part of the right thyroid gland, with an uneven internal echo, irregular shape, and unclear boundary. **(C)** Shear-wave elastography showing the hypothyroid area on the right as the essentially blue area. **(D)** Color Doppler ultrasonography showing minimal blood flow signal in the hypoechoic thyroid area.

**Figure 3:**
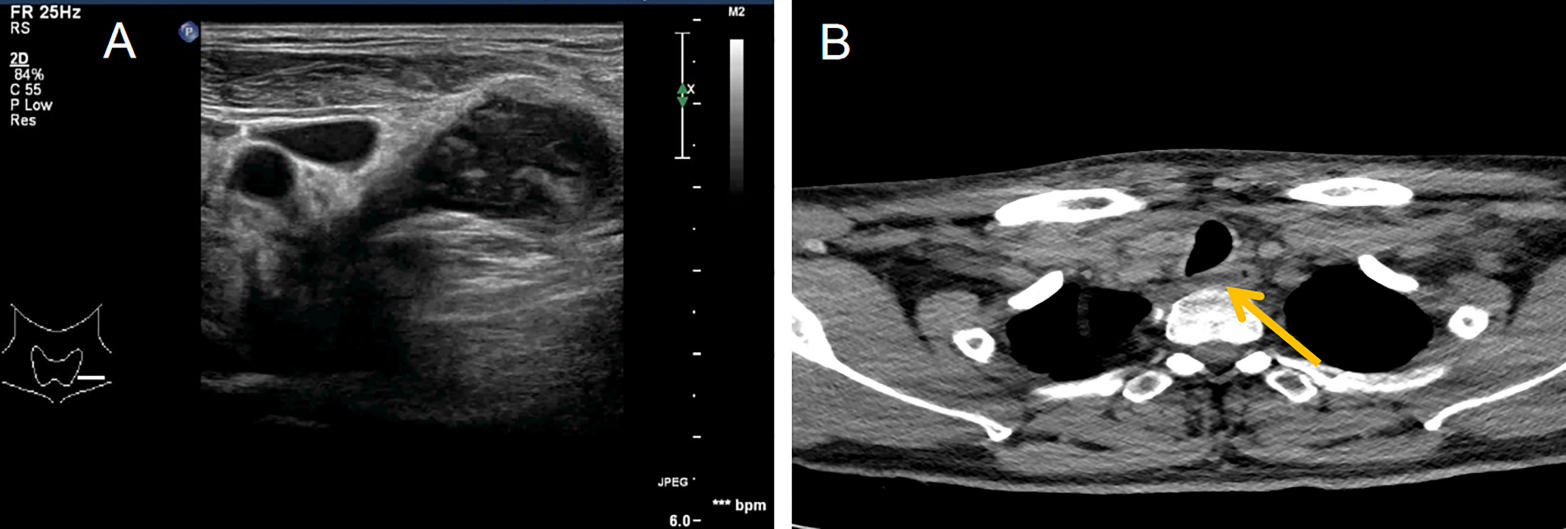
Ultrasound and computed tomography showing the posterior neck. **(A)** Ultrasound showing mixed echoes on the left side of the neck, poorly demarcated from the surrounding soft tissue and appearing to extend deep into the neck. This presentation is similar to the formation of tuberculous sinus passages in lymph nodes. **(B)** Neck computed tomography showing streaky fluid density shadows in front of the cone (yellow arrow points).

**Figure 4:**
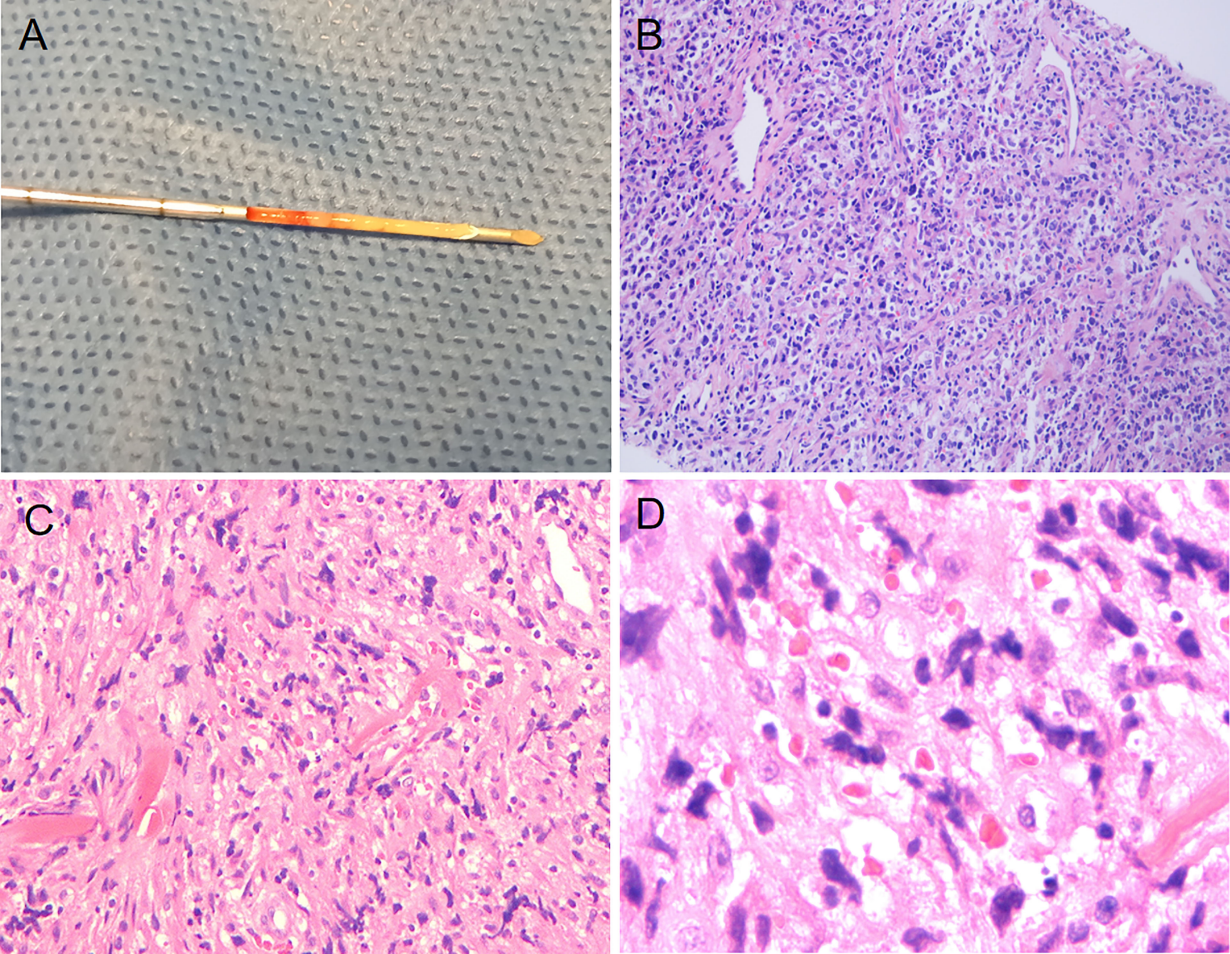
Puncture specimen obtained through ultrasound-guided puncture and histopathologic images showing cells with pleomorphism. **(A)** Puncture specimen of the right cervical lymph node (obtained from the Hangzhou Red Cross Hospital). **(B)** Pathological images taken with an optical microscope at 400× magnification (right cervical lymph node, obtained from the Department of Pathology at Hangzhou Red Cross Hospital). **(C)** Pathological images taken with an optical microscope at 10× magnification (left cervical lymph node, obtained from the Department of Pathology at Zhejiang Cancer Hospital). **(D)** Pathological images taken at 40× magnification under an optical microscope (left cervical lymph node, obtained from the Department of Pathology at Zhejiang Cancer Hospital).

A 43-year-old Chinese male patient underwent ultrasonography at a local hospital because of swelling in the neck and face for 1 week. The patient complained of no pain in the neck and had no other symptoms except occasional respiratory disturbances. He was previously healthy and had no known medical conditions. There was no history of fever/cough, appetite loss, or weight loss. Ultrasound examination of the thyroid gland and bilateral cervical lymph nodes was performed at a local hospital, which suggested tuberculosis. The patient did not receive any medical treatment at the local hospital. Following this, the patient visited Hangzhou Red Cross Hospital for further examination. During the examination, multiple enlarged lymph nodes with clear margins and uneven internal echoes were found in the neck; lymphatic hilum blood flow could be noted; and the lymph nodes appeared mostly blue on ultrasound shear-wave elastography, indicating a firm texture (**Figure 1**). A striate hypoecho was observed in the middle and lower right lobe of the thyroid gland, suggesting thyroid invasion (**Figure 2**). Simultaneously, a mixed-echo mass indistinguishable from the surrounding soft tissue was found upon imaging of the left neck, which resembled the formation of a sinus following the rupture of lymph node tuberculosis; abnormal echoes were also present in front of the cervical spine (**Figure 3A**). We considered that the patient most likely had tuberculosis, either cervical vertebra tuberculosis or lymph node tuberculosis, considering that the mixed echoes of the neck reflected lymph node formation in a tuberculosis sinus tract. Thus, cervical spine computed tomography (CT) was performed immediately. However, the results showed no obvious abnormalities in the cervical cone or bone damage. A density of patchy fluid was observed on the posterior wall of the larynx (anterior cone of the cervical spine; **Figure 3B**). In the afternoon of the same day, we performed contrast-enhanced ultrasound (CEUS) evaluation, which revealed a large mixed-echo mass (areas III and IV) in the left neck, which rapidly filled with internal contrast agent and showed high enhancement at the peak. No nonenhancement areas were found. A cervical lymph node in area III, which was 3.0 × 1.6 cm in size, suggested a rich blood supply and exhibited centripetal and high enhancements at the peak (**Figure 1**). Therefore, an ultrasound-guided puncture biopsy (with an 18-G core needle was performed (**Figure 4A**). The mixed-echo mass on the left side and one enlarged lymph node on the right side were punctured. Eight biopsy samples were collected from each site, and 2-cm long samples were sent for pathological examination, which suggested that the left neck mass was a quasi-circular malignant tumor with partial crush injury; thus, lymphoma was considered a possible diagnosis. The pathologic diagnosis of the right cervical lymph node sample was round-cell malignant tumor with focal coagulative necrosis (**Figure 4B**). Additional tests for tuberculosis, including acid-fast staining and the Xpert^®^ test, were negative.

After excluding tuberculosis as a diagnosis, the patient was admitted to Zhejiang Cancer Hospital for specialized treatment. A relevant laboratory examination was completed after admission. The assessment of thyroid function revealed only an elevated level of antithyroid peroxidase antibodies (219.1 U/mL). Hypersensitive C-reactive protein level was 13.42 mg/L, which was slightly higher than the normal range. Since the disease onset, the patient exhibited a good clinical picture, with regular sleep pattern and no significant weight loss. In addition, the patient did not consume alcohol or tobacco products, had a harmonious family relationship, and did not have a familial genetic disease history. The patient has been living in Hangzhou, Zhejiang Province, China for a long time since birth and has no family history of tuberculosis; he was vaccinated against tuberculosis in accordance with the national regulations. CT before chemotherapy indicated the invasion of the superior vena cava (SVC). Furthermore, the patient’s breathing was irregular. The patient underwent a second puncture biopsy using an 18-G core needle, and pathological analysis showed only the puncture result of one lymph node in the left neck. The tissue was fusiform, suggesting diffuse large B-cell lymphoma (**Figures 4C, D**).

**Figure 5:**
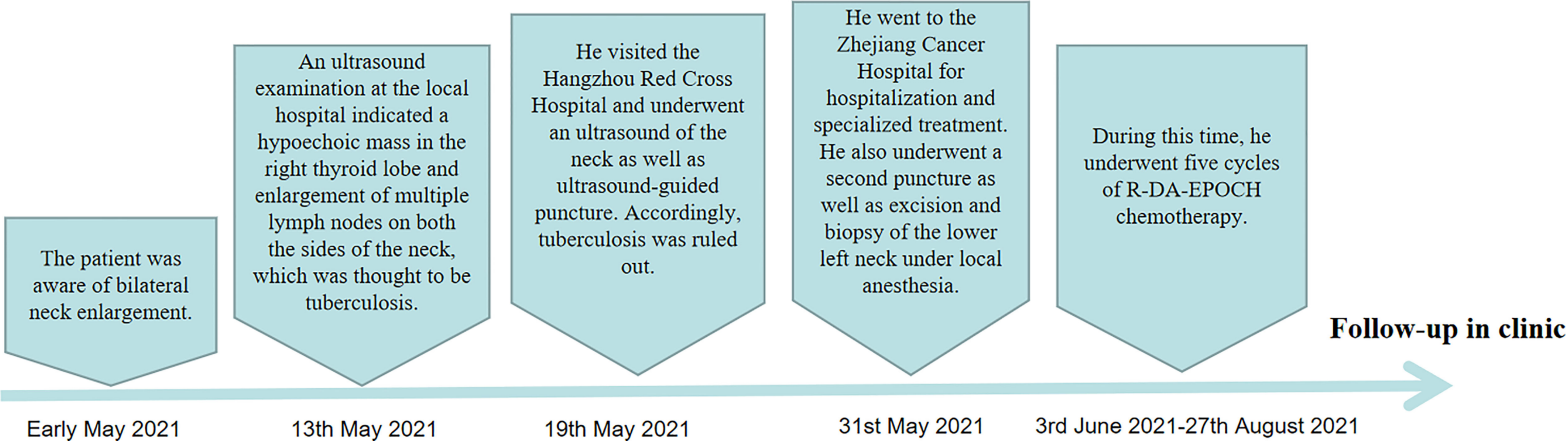
The flowchart from disease discovery to diagnosis and treatment of the patient.

Although the physical and additional clinical examinations suggested lymphadenopathy and potential lymphoma, the pathological classification was unclear. With the consent of the patient, surgical biopsy was performed, which led to the pathological diagnosis of PMBCL. At the last follow-up, the patient had completed the fifth cycle of chemotherapy (R-DA-EPOCH), with a favorable outcome. At present, he has no neck pain, nausea, fatigue, or other symptoms, and the swelling in the neck had resolved. Thyroid lesions had also improved. Informed consent was obtained for all invasive procedures performed in this case. The flowchart from disease discovery to diagnosis and treatment of the patient is shown in the **Figure 5**.

## Discussion

PMBCL is classified as a separate disease entity by the World Health Organization, and typical histologic presentations include a diffuse infiltrate of centroblasts, immunoblasts, multilobated cells, and/or Reed-Sternberg–like cells often associated with a delicate nesting vesicular pattern or with coarse sclerosis. These findings overlap with those for classical nodular sclerosing Hodgkin’s lymphoma (6–9), thereby making the diagnosis of PMBCL difficult. Therefore, the initial pathological and immunohistochemical findings were considered reflective of Hodgkin’s lymphoma.

Pathologic and immunohistochemical findings at Zhejiang Cancer Hospital showed CD20(+), CD30(+, 80%), CD23(−), EBER(−), c-Myc(+, 40%), bcl-2(+, 60%), which indicated aggressive large B-cell lymphoma. However, owing to insufficient puncture tissue, the specific classification of lymphoma could not be determined. Later, a lymph node in the left lower neck was excised and biopsied under local anesthesia, and pathological immunohistochemistry and second-generation sequencing indicated PMLCL.

PMLCL often occurs in young people, particularly in women. However, in the present case, the patient was a middle-aged man. The most common clinical presentation of PMBCL (more than two-thirds of cases) is a large anterior mediastinum mass with local tumor compression, leading to rapidly progressive symptoms, such as dyspnea, cough, dysphagia, compromised airway and great vessels, and SVC obstruction. Lymphoma is similar to tuberculosis to some extent in clinical presentation. Nevertheless, CT indicated SVC invasion in the current case and evidently altered breathing was noted prior to chemotherapy. The patient in this case had enlarged cervical and mediastinal lymph nodes as well as thyroid lesions, which had to be differentiated from tuberculosis, Kimura disease, pulmonary malignant tumor, sarcoidosis, and lymph node hyperplasia. Although the imaging of these diseases is similar, the diagnosis of these diseases is always primarily dependent on histopathology.

Freeman et al. (10) reported that extranodal manifestations of lymphoma occur in 20%–30% of patients. Although the distant spread of PMBCL is rare at the time of diagnosis, it tends to disseminate to extranodal sites, such as the kidneys, ovaries, central nervous system, gastrointestinal tract, and liver (9). To the best of our knowledge, cases where PMBCL spread to the thyroid gland have not been reported yet. In the present case, the right thyroid ultrasound showed a low-echo area with an unclear boundary and poor blood flow signal. Yang et al. (5) described a case of thyroid tuberculosis where the ultrasonic findings were similar to those in the present case to some extent, specifically, the unclear boundary and heterogeneous, low-echo area in the thyroid. Furthermore, owing to the rare extranodal ultrasound findings of the lymphoma and the patient’s previous thyroid ultrasound examination showing no abnormalities, our diagnosis at that time was more inclined to tuberculosis considering the patient’s lymph node ultrasound. In addition, the patient’s neck was compressed and injured due to the compression from lymph node enlargement, resulting in abnormal echo in the soft tissue around lymph nodes and thyroid invasion. When sinus lymph node tuberculosis occurs, the surrounding soft tissue also changes on ultrasound, which easily leads to a misdiagnosis. Ahuja et al. (11) found no significant differences in the ultrasonic characteristics of different pathological subtypes of lymphoma.

The literature indicates that CEUS is a promising diagnostic modality to differentiate between benign and malignant lymph nodes (12). CEUS provides information on vascularization and perfusion patterns and visualizes the differences in blood flow characteristics between normal and pathological tissues. However, the evaluation of lymph nodes provides insufficient diagnostic information (13). Our examination found the patient’s enlarged lymph nodes showed a rapid enhancement mode on CEUS. Ma et al. (14) suggested that lymphomatous lymph nodes can be classified into two types based on CEUS, which may be used as differential diagnostic criteria: rapid and uniform high enhancement, with 83.1% of lesions showing rapid enhancement mode in arterial phase, and rapid and uneven high enhancement, with 16.9% of lesions showing uneven enhancement in arterial phase. Lymph node tuberculosis may also show uniform enhancement (15, 16), and the internal sonographic manifestations of lymph node tuberculosis are closely related to pathological changes (12, 17, 18). Therefore, tuberculosis could not be ruled out even after performing CEUS in the present case; thus, puncture biopsy was performed to confirm the diagnosis.

Shear-wave elastography is a recent technological advancement in ultrasonography that enables the assessment of tissue stiffness (19). The main pathological change of lymphoma is the clonal proliferation of single tumor cells. Therefore, more primitive lymphocytes, fewer stromal cells, and lower elasticity are observed (20). PMBCL is pathologically similar to nodular sclerosing Hodgkin’s lymphoma because of connective tissue hyperplasia, which increases the hardness of lymphomas (21). In the present case, shear-wave elastic ultrasound of enlarged lymph nodes and thyroid lesions showed a hard endoplasm, which might have resulted from their pathological characteristics.

Ultrasound is a safe, real-time diagnostic tool that is preferred to evaluate lymph nodes and plays a significant role in disease prognosis (22, 23). Ultrasound-guided needle biopsy is a safe, quick, and valid tool for diagnosing lymphadenopathy (24). However, the use of needle aspiration in the diagnosis of lymph node disease remains limited (25, 26). In this case, the patient underwent two needle biopsies, both of which resulted in a lymphoma diagnosis, but no specific classification could be determined. To determine the pathologic classification, a surgical biopsy was eventually needed. Although the type of puncture needle used in both puncture biopsies was the same, it was unclear whether the amount of tissue collected was the same in both puncture biopsies. Therefore, the inconsistent results between the two puncture biopsies might be because of the insufficient amount of tissue collected during the puncture biopsies, different lymph nodes that were biopsied by puncture, and atypical lesions of some lymph nodes.

History, physical examination, radiographic studies, laboratory tests, and initial biopsy pathology with immunohistochemistry are used to diagnose and stage PMLBCL (27). X-rays, CT scans, and MRI scans can also be used to diagnose and stage PMLBCL. On cervicothoracic radiographs or CT, anterior mediastinal masses are the most common finding and tracheal retroflexion with or without stenosis can occur. Unilateral diaphragmatic elevation, pleural effusion, and pericardial effusion are further radiographic features (28). When there is a residual mass after treatment, an MRI can be helpful. On T2-weighted images, residual tumors have heterogeneous signal intensity. T2-weighted images with high signal intensity or T1-weighted images with low signal intensity may indicate residual active lymphoma, necrosis, or other conditions. Fibrotic masses exhibit homogeneous hypo-intensity (29).

Todeschini et al. conducted a retrospective study on 138 patients and found that patients with PMLBCL who were treated with third-generation regimens, such as etoposide/methotrexate, doxorubicin, cyclophosphamide, vincristine, prednisone, and bleomycin, had higher response rates and longer depth-free survival and overall survival than those who were treated with regimens based on Cyclophosphamide, Doxorubicin, Vincristine, and Prednisone (CHOP) (30). Rituximab with CHOP improved outcomes for all DLBCL subtypes, including PMLBCL (31). The R-DA-EPOCH chemotherapy regimen was employed in this case.

Although lymphoma is very common in clinical practice, it is often misdiagnosed, causing delayed treatment initiation and affecting patient outcomes as the disease progresses. The present case demonstrates that the sonographic appearance of lymphoma may sometimes be confused with that of tuberculosis. Although ultrasound-guided needle biopsy has a high diagnostic accuracy, it may also lead to diagnostic deviation because of insufficient sampling volume. Moreover, owing to the enlargement of multiple lymph nodes due to lymphoma or lymph node tuberculosis, puncturing different lymph nodes may present different results.

## Data Availability Statement

The original contributions presented in the study are included in the article/supplementary material. Further inquiries can be directed to the corresponding author.

## Ethics Statement

Written informed consent was obtained from the individual(s) for the publication of any potentially identifiable images or data included in this article.

## Author Contributions

YW: manuscript writing. MC: data collection. CN: data collection. YZ: data collection. PC: data collection. JT: data collection. GY: supervision and data collection. All authors contributed to the article and approved the submitted version.

## Funding

This work was supported by the Agriculture and Social Development Plan of Hangzhou [grant number 20190101A09] and the Medical Science and Technology Project of Zhejiang Province [grant number 2021KY911].

## Conflict of Interest

The authors declare that the study was conducted without any business or financial relationships that could be interpreted as a potential conflict of interest.

## Publisher’s Note

All claims expressed in this article are solely those of the authors and do not necessarily represent those of their affiliated organizations, or those of the publisher, the editors and the reviewers. Any product that may be evaluated in this article, or claim that may be made by its manufacturer, is not guaranteed or endorsed by the publisher.
